# Supplementary material for: Splice-Junction-Based Mapping of Alternative Isoforms in the Human Proteome
Source: Cell Rep. Author manuscript; Available in PMC 2020 Jan 15. (PMC6961840; doi:10.1016/j.celrep.2019.11.026)
Supplement: 2 [file NIHMS1546469-supplement-2.zip › DF1/PXD006675/RightVentricle/RightVentricle_10_TTN_TPVQEEVIEVKVPEVPK.pdf]

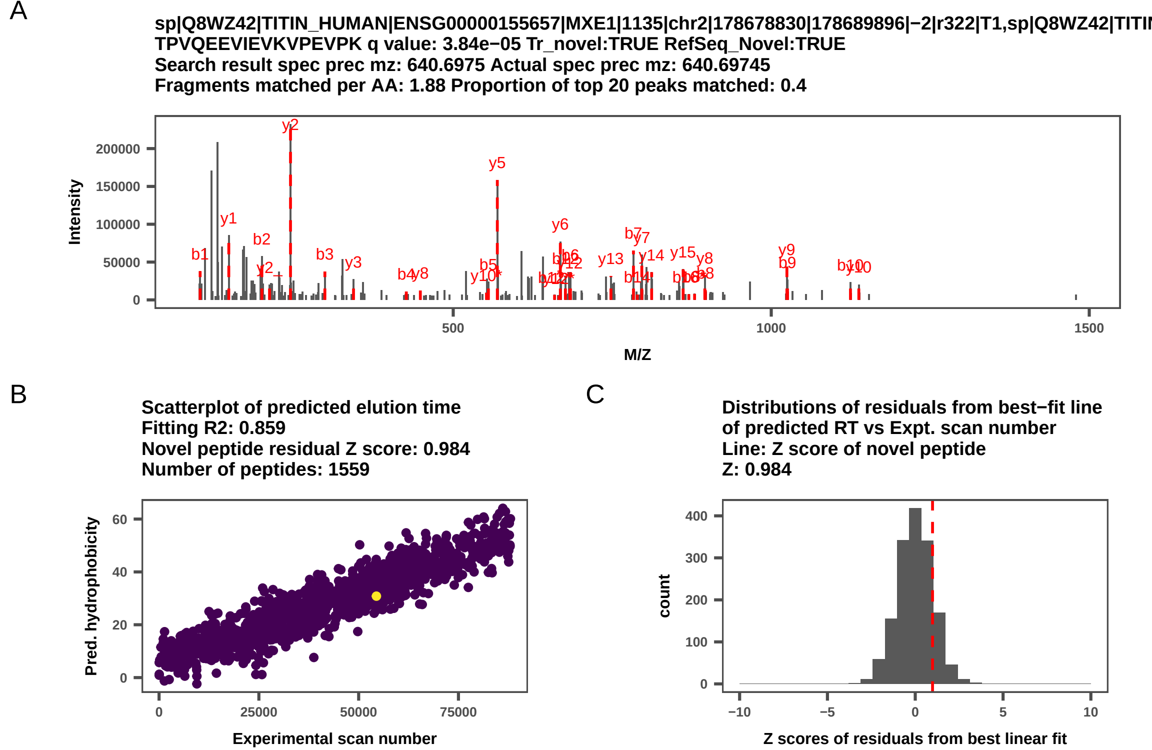

A
sp|Q8WZ42|TITIN_HUMAN|ENSG00000155657|MXE1|1135|chr2|178678830|178689896|−2|r322|T1,sp|Q8WZ42|TITIN
TPVQEEVIEVKVPEVPK q value: 3.84e−05 Tr_novel:TRUE RefSeq_Novel:TRUE
Search result spec prec mz: 640.6975 Actual spec prec mz: 640.69745
Fragments matched per AA: 1.88 Proportion of top 20 peaks matched: 0.4
Intensity
M/Z
B
Scatterplot of predicted elution time
Fitting R2: 0.859
Novel peptide residual Z score: 0.984
Number of peptides: 1559
Pred. hydrophobicity
Experimental scan number
C
Distributions of residuals from best−fit line
of predicted RT vs Expt. scan number
Line: Z score of novel peptide
Z: 0.984
count
Z scores of residuals from best linear fit
